# Exploring the barriers to optimal survivorship care for people living with Cancer in NSW

**DOI:** 10.1007/s00520-026-10921-6

**Published:** 2026-07-01

**Authors:** Yuqi Ouyang, Helen Tran, Brad Gellert, Elizabeth Kennedy, Carolyn Mazariego-Jones, Michael David, Janette Vardy

**Affiliations:** 1 Policy and Advocacy Unit, Cancer Council NSW, Sydney, NSW Australia; 2 School of Population Health, UNSW, Kensington, NSW Australia; 3 The Daffodil Centre, University of Sydney, a joint venture with Cancer Council NSW, Sydney, Australia; 4 School of Medicine & Dentistry, Griffith University, Southport, QLD Australia; 5 Sydney Cancer Survivorship Centre, Concord Cancer Centre, Concord Repatriation & General Hospital, Hospital Rd, Concord, NSW 2139 Australia; 6 Faculty of Medicine and Health, University of Sydney, Camperdown, NSW Australia

**Keywords:** Cancer survivorship, Supportive care, Policy advocacy, Optimal cancer survivorship

## Abstract

**Purpose:**

The study aims to understand the perceptions and provision of survivorship care among cancer survivors and identify barriers to optimal care. The findings will also provide insights to help Cancer Council NSW to develop policy recommendations to ensure survivors receive comprehensive support throughout their post-treatment journey.

**Methods:**

A cross-sectional survey was conducted among cancer survivors aged 18 and older who had completed active treatment (*n* = 209). Data on survivorship care experiences, barriers to access, and unmet needs were collected. Firth logistic and ordinal regression models were used to analyse associations between outcomes (ease of accessing care, information about supportive care, care effectiveness, and unmet needs) and demographic, socioeconomic, and clinical predictors.

**Results:**

Most patients (64.2%) received information about supportive care services, and 72.2% felt their needs were met, but only 44.8% found access easy. Educational level was significantly associated with the effectiveness of care after treatment; while lacking a survivorship care plan was associated with lower perceived effectiveness of care after treatment (adjusted OR: 12.80, *P* < 0.001). Women reported significantly greater difficulty accessing supportive care services (adjusted OR: 0.08, *P* = 0.004) compared to men. Key barriers included high costs and long appointment wait times, particularly for psychological support, exercise physiology and specialist care.

**Conclusion:**

Addressing barriers to survivorship care in NSW requires targeted policy interventions, including financial support, enhanced access across gender groups, and standardised survivorship care plans.

**Implications for Cancer Survivors:**

The findings assist Cancer Council NSW advocate for policy recommendations to enhance survivorship care services, ensuring equitable access for all cancer survivors.

**Supplementary Information:**

The online version contains supplementary material available at 10.1007/s00520-026-10921-6.

## Introduction

Advancements in cancer treatment have improved survival rates, resulting in the exponential growth in cancer survivors worldwide. Over 15.5 million people were estimated to be living with a cancer history in 2016 and projections suggesting this figure would rise to 20.3 million by 2026 [1]. In Australia, cancer survivorship is increasingly recognised as a major public health issue. Based on research from Cancer Council New South Wales (CCNSW), it is predicted that more than 1.5 million people in New South Wales (NSW) will be diagnosed with cancer between 2020 and 2044 [2]. As more people live beyond cancer, attention has shifted particularly in the provision of long-term survivorship care [1].

The Clinical Oncology Society of Australia (COSA) defines a cancer survivor as anyone who has been diagnosed with cancer for the remainder of their life [3]. However, the COSA model of Survivorship Care specifically focuses on care following the completion of primary treatment, when survivors transition from active treatment to follow-up care [3]. Consistent with this model, this study focused on post-treatment survivorship care, as the transition from active treatment to survivorship is often complex, with many individuals facing long-term physical, psychological, and social challenges that are not always adequately addressed by existing healthcare systems [4]. Traditional surveillance-focused follow-up models often inadequately address long-term treatment side effects, chronic comorbidities, and lifestyle-related needs.[4]. As a result, the provision of optimal survivorship care has become increasingly essential to address the continuous needs of cancer survivors throughout their cancer journey.

In Australia, optimal survivorship care models aim to enhance cancer care through a multi-disciplinary approach emphasising patient-centred strategies [3]. The model developed by COSA identifies principles that address survivors’ multifaceted needs, encompassing physical and psychosocial aspects of life post-diagnosis and treatment, and through a risk-stratified follow-up care pathway, ensuring tailored support and coordination between oncology, primary care, and allied health providers [3]. In practice, survivorship care plans (SCPs) are a key component of supporting cancer care continuum, providing patients with a comprehensive summary of their diagnosis, treatment, follow-up care, and strategies for managing long-term effects [3, 5]. Several Australian states have additionally developed survivorship frameworks and cancer plans, emphasising that supportive care should be holistic and patient-centred, safe, accessible, effective, efficient, and equitable [5–7]. The strategies outlined in these frameworks and plans ensure that survivorship care not only targets the disease but also promotes the overall health and well-being of individuals.

Despite several Australian survivorship frameworks and policies being developed to guide optimal survivorship care, gaps remain between policy recommendations and real-world implementations. The current literature highlights significant patient and health system barriers to achieving optimal survivorship care. Many patients, particularly those in remote and rural areas, face limited access to healthcare services [8, 9]. The financial burden of out-of-pocket payments often makes essential follow-up care unaffordable, forcing patients to often seek guidance from non-traditional sources [9, 10]. This complicates their ability to navigate the healthcare system, as well as to effectively manage the physical and psychosocial consequences of cancer and its treatment. Previous research has also highlighted the importance of age and gender in the understanding of cancer dynamics across different populations. Age influences biological and physiological changes that can determine the types of supportive care services offered, as older patients might require unique forms of support compared to younger ones, affecting both the accessibility and relevance of the services provided [11]. Gender influences how support is considered and used, as cultural norms and expectations can impact whether individuals seek and get appropriate care [12]. Additionally, culturally and linguistically diverse (CALD) patients are not adequately accommodated for in survivorship care provision [13]. The dominance of the biomedical model marginalises alternative therapies [14]. Aboriginal and Torres Strait Islander people continue to face challenges related to the ongoing impacts of colonisation, systemic racism, and limited cultural safety within healthcare settings, including experiences where cultural identities, values, and needs are not adequately recognised or respected [15]. These challenges foster mistrust towards the healthcare system and cultural misunderstanding among healthcare providers. At the same time, long wait times and a mismatch between available and needed services delay critical care, impacting recovery and quality of life, and a lack of provider training in cancer survivorship, which weakens the continuity and effectiveness of care [8–10]. Addressing barriers related to cost, accessibility, cultural misunderstanding, and healthcare coordination is essential to ensuring that all cancer survivors receive timely, accessible, and comprehensive support for their long-term health and well-being.

Although many survivorship care barriers have been described internationally and across Australia, there remains limited NSW-specific evidence examining cancer survivors’ experiences of survivorship care, perceptions of care effectiveness, and barriers to accessing supportive care services. Understanding these barriers within the NSW healthcare context is important for informing state-level advocacy and policy development, particularly given differences in healthcare delivery, service accessibility, and resource distribution across states. Therefore, this research project aims to explore the current condition of survivorship care, identify the barriers to optimal survivorship care, and identify the specific needs of cancer survivors in NSW.

## Method

### Study design

The study utilised a cross-sectional study design consisting of an online survey to collect data from a target group of cancer survivors aged 18 and above. After completing the demographic details, participants were divided into two groups based on their cancer diagnosis: those with a recent cancer diagnosis or who were currently undergoing active cancer treatment and those with a past cancer diagnosis (completed active treatment). Each group completed a separate survey to capture the unique experiences of patients.

### Participants and recruitment

Recruitment was conducted over approximately ten months with the aim of collecting as many responses as possible during this period. Recruitment strategies included direct email invitations to CCNSW's online mailing lists and CanAct Community, an advocacy network of people affected by cancer who support CCNSW’s policy and advocacy activities. Invitations to participate were also promoted through networks and stakeholders, including Cancer Voices NSW, Breast Cancer Network Australia, Prostate Cancer Foundation of Australia, and Health Consumers NSW. A passive snowball sampling method was used, allowing existing participants to refer to potential participants [16]. To encourage participation, the study offered an entry into a draw for a $100 supermarket e-gift card.

The survey was initially designed to capture experiences across different stages of the cancer care continuum, including participants with current or past cancer diagnoses. However, aligning with the COSA model of Survivorship Care focusing on survivorship care beginning after the completion of primary treatment, only participants who had completed their primary treatment were included in the final analysis. The survey for this group concentrated on their experiences in survivorship care, including their needs during this phase and the barriers they encountered in accessing optimal survivorship care.

### Consent

All necessary information, including consent forms and demographic questions, was accessed and completed online, ensuring participants were fully informed and their eligibility confirmed at the beginning of the study. Participation in the study was voluntary, and consenting participants could withdraw at any time.

### Data collection and management

All data were collected from participant responses, including consent forms, demographic details, and survey answers, and were stored securely within Australia in accordance with ethical and legislative requirements. Data were managed under Australian Code for the Responsible Conduct of Research and CCNSW’s Information Security Policy, with electronic data stored on secure networks and SharePoint, and paper records kept in locked, access-controlled areas. Identifying information was stored separately from survey data using alphanumeric ID codes. After five years, all data will be securely deleted or destroyed. Non-identifiable data may be shared with approved collaborators through secure platforms, with CCNSW retaining data custody.

### Variables

#### Outcomes of interest

To investigate the research question, four outcome measures were assessed through survey data collection:

1. Information about supportive care services: Participants were asked if they had received information about supportive care services and to identify the source of the information. For analysis, receiving information from a hospital or treating team, a General Practitioner (GP), family or friends, online sources, or other unspecified sources was coded as “yes” [17]. Conversely, responses indicating no information or no awareness of supportive care services were coded as “no”.
2. Ease of access to supportive services: Participants rated how easy to access support services on a five-point scale ranging from “Very easy” to “Very difficult”. Responses with “Neither/I did not need to access the service” were considered missing in subsequent analysis.
3. Perceived effectiveness of care after treatment: Similarly, this was assessed on a five-point scale, from “extremely effective” to “not at all effective”.
4. Support met the needs of patients: Participants were asked whether they felt the support met their needs, with options “yes” and “no”.

#### Predictors

Explanatory variables were structured into three groups: demographic, socioeconomic, and clinical.

The demographic variables included age, gender (woman, man, and non-binary), Aboriginal and Torres Strait Islander (yes, no), country of birth (Australia, outside of Australia), and place of residence. The variable “place of residence” was derived from the postcode provided by participants. Using the Health Workforce Locator, which applies the Modified Monash Model (MMM) geographic classification system. Locations categorised as MM1 were classified as “metropolitan”, while locations classified as MM2–MM7 were grouped as “regional” [18].

The socioeconomic variables included educational level (primary/secondary school, certificate or diploma, undergraduate degree, postgraduate degree) and employment status (non-retired, retired).

The clinical variables included type of cancer, stage of cancer, and whether patients received written or digital SCPs.

Employment status was categorised as retired versus non-retired, and type of cancer was grouped as breast cancer versus non-breast cancer, given the relatively small sample size and sparse observations across several subgroups. These broader classifications were applied to improve interpretability and support more stable regression estimates. Stage of cancer was divided according to the Cancer Institute NSW [19] into “Stage 0: cancers are found in the place they started. They have not spread.” “Stage 1: the cancer has not grown deeply into nearby tissues. It has not spread to the lymph nodes or other parts of the body. It is often called early-stage cancer.” “Stage 2 & 3: cancers that have grown deeply into nearby tissue. It may have spread to lymph nodes but not to other parts of the body.” “Stage 4: cancer has spread to other organs or parts of the body. It may also be called advanced or metastatic cancer.” Questions were also included to identify the most common healthcare professionals that participants need to see but are unable to, and the top reported barriers they face in accessing these healthcare professionals. The responses were then counted to determine the most frequently mentioned items in each variable.

In the dataset, the categories “I do not know” or “Prefer not to say” were included in the survey for each variable. During data analysis, responses falling into these categories were treated as missing data to ensure the analysis remains robust by excluding uncertain or unspecified data.

### Statistical analysis

Categorical variables were summarised using counts and relative frequencies for categorical variables. To enhance clarity in the table presentation, age was grouped into quartile-based categories (< 60, 60–65, 66–74, and > 74) in Table 1 to maintain adequate numbers within each category. Due to non-convergence issues caused by the small sample size, Firth logistic regression modelling [20] was used in conjunction with a complete case approach to analyse the dichotomous dependent variables “whether received information about supportive services”, and “whether support met patients’ needs” (1 = yes; 0 = no), due to a small sample size. Ordinal logistic regression models were also performed to assess “Easiness to access supportive services” and “Effectiveness of care after treatment”. These models allowed for the analysis of ordinal outcome variables, capturing the ordered categories of the survey responses while adjusting for relevant covariates. Univariable logistic regression analyses provided crude odds ratios (ORs) and their 95% confidence intervals (CIs). This method addresses the issue of bias in maximum likelihood estimates that can occur with limited data in categorical variables, ensuring more reliable and accurate model estimation [21]. This is especially valuable in cases where the sample size is insufficient for certain categories. Age and gender were forced in the multivariable models. Other variables that demonstrated significance at the 0.10 level in univariable analyses were examined for potential impact on the outcome in the final multivariable model.

Before completing the multivariable model, a check for collinearity among the variables was conducted to prevent issues such as unstable or biased estimates, which could also result in nonconvergence [22]. In the final multivariable model, the adjusted ORs with 95% CIs were presented for all included variables, and statistical significance was determined using a threshold of *P* < 0.10 for univariable analysis and *P* < 0.05 for multivariable analysis. All statistical analyses were conducted by RStudio Version 4.4.0.

## Results

**Fig. 1:**
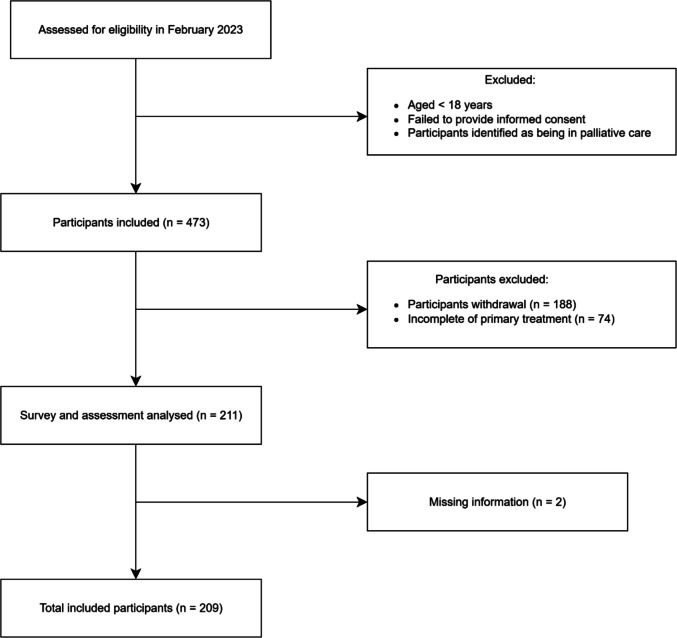
STROBE flow chart summarising the studying selection and assessment process

In total, 473 individuals identified as potential participants based on predefined eligibility criteria started the CCNSW’s Survivor Survey. Of these, 209 met the requirements and completed the survey, resulting in a final completion rate of 44.2% (Fig. 1) (Appendix 1).

As reported in Table S1, participants ranged in age from 24 to 88 years, with the largest proportion (26.1%) in the 66–74 age group. Most respondents were women (*n* = 151, 72.2%), and only 1.9% identified as Aboriginal and Torres Strait Islander. Overall, 54.6% (*n* = 113) of the participants resided in metropolitan areas, while 45.4% (*n* = 94) lived in regional areas. The educational background varied, with 61 participants (29.8%) holding a postgraduate degree, approximately a quarter (*n* = 56) having a university degree, 30.7% holding certificates or diplomas, and 12.2% (*n* = 25) having only primary or secondary education. Employment status was almost evenly split with 51.5% still working and 48.5% retired. The most reported cancer type was breast cancer (39.3%), and the cancer stage distribution showed that 51.5% (*n* = 87) were diagnosed at stage 2 and 3.

**Fig. 2:**
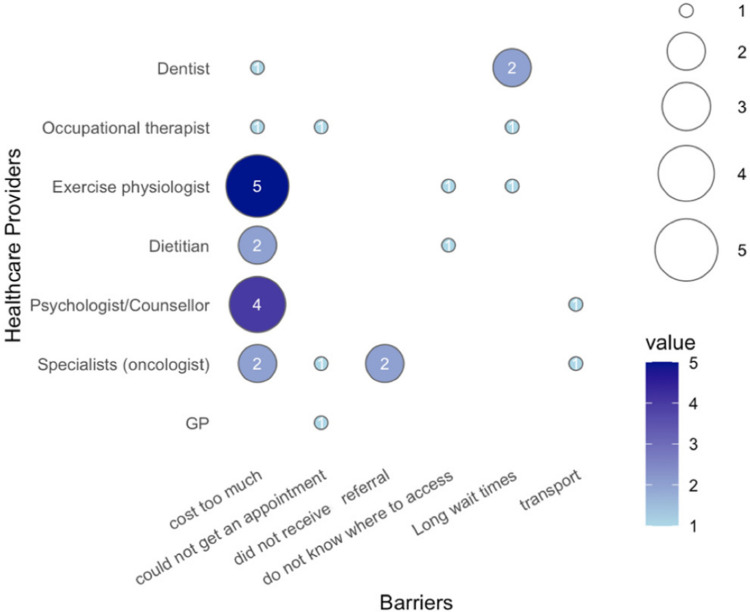
Most inaccessible healthcare professionals and top reported barriers preventing patients accessing them

Only 10.9% of participants reported receiving a written or digital SCP. Despite this, 64.2% of respondents received information about supportive care services, and 72.2% stated that the support provided met their needs. However, barriers to accessing supportive care services were frequently noted, with 40.4% of participants finding it “a little difficult” and 14.7% finding it “very difficult” to access such services. The most reported unmet healthcare needs included access to exercise physiologists (31.6%), psychologists (26.3%), and specialists (26.3%). The top barriers preventing access to necessary healthcare services were cost (38.9%) and long wait times (27.8%). It is important to note that the total number of responses for both these questions was limited, with only 19 participants answering the questions about healthcare professional needs and 12 participants answering the question about barriers (Fig. 2).

Tables S2 and S3 show no significant associations between the outcome variables (whether patients received supportive care services and whether the support met their needs) and the potential predictors after adjusting for age and gender.

Table 1 examines the potential factors influencing the effectiveness of care after treatment. Educational level was significantly associated with the effectiveness of care after adjustment. Specifically, those with a postgraduate degree (OR: 18.67; 95% CI: 3.21, 132.54; *P* = 0.002), undergraduate degree (OR: 7.33; 95% CI: 1.09, 57.93; *P* = 0.046), and certificate or diploma (OR: 12.26; 95% CI: 2.07, 87.61; *P* = 0.008) were significantly less likely to report effectiveness compared to those with only primary or secondary education. In addition, no significant association was found to exist between the receipt of a survivorship plan (written or digital) and effectiveness of care (OR: 12.80; 95% CI: 0.21, 65.27; *P* < 0.001).

Gender showed a significant association with the ease of accessing supportive care services, as shown in Table 2, with women being more likely to experience difficulties accessing these services than men (OR: 0.008; 95% CI: 0.01, 0.42; *P* = 0.004).

## Discussion

This study provides valuable insights into the current state of survivorship care for cancer patients in New South Wales, identifying significant barriers that need to be addressed to optimise care delivery. The findings suggest the need for targeted improvements in access and the quality of survivorship care.

Overall, 64.2% of participants reported receiving information about supportive care services, with hospitals, treating teams, General Practitioners (GPs), family, friends, and online sources being the primary channels according to the survey. While this suggests that most survivors have some awareness of available support, the remaining 35.8% who did not receive such information highlights a critical gap in survivorship care. Similarly, research in Finland found that about one third of patients reported receiving little or very little information about rehabilitation, communication with family and friends, and available financial support [23]. However, no significant predictors were found in this study to influence the outcome variable, suggesting that factors influencing this may not be captured in this study, such as individual preferences, provider availability, or systemic healthcare inequalities.

Even for those who were aware of supportive care services, accessibility remains a significant issue, with nearly 55% of participants reporting difficulties in accessing supportive care services. Several studies also reported difficulties related to accessibility, including limited service availability, fragmented care coordination, and delays in accessing allied health support [24, 25]. These findings suggest that the access difficulties reported by participants in the current study are not unique to NSW, but reflect broader challenges in implementing coordinated and accessible survivorship care. This can be attributed to systemic barriers such as long wait times, high out-of-pocket costs, and the limited availability of specialised healthcare providers. These barriers can lead to fragmented care and missed opportunities for early intervention in managing long-term treatment effects.

Financial constraints were among the most mentioned barriers to accessing survivorship care, with nearly 40% of participants reporting that cost prevented them from seeing necessary healthcare providers. Supportive care services, including those provided by exercise physiologists and psychologists, often lack full coverage by Medicare or private health insurance, making them unaffordable for some survivors. A study from the Daffodil Centre revealed that over 50% of people with cancer in the past two years reported spending more than AUD 1,000 from their own pockets, with nearly one in ten facing expenses over AUD 10,000 [26].

The financial burden encompasses not only the direct expenses of treatments such as medications and procedures but also indirect costs like transportation, lost income, and the need for specialised care [27]. These financial burdens can lead to stress and poor adherence to treatment protocols. Twenty percent of men with prostate cancer reported that the cost of their treatment caused significant distress [28], highlighting the pressing need for financial assistance programs in survivorship care. Telehealth has shown promise in reducing the financial burden and logistical burden associated with cancer care by minimising transport costs, reducing income loss due to time away from work, and improving access to supportive care services [29–31]. In addition, existing financial assistance programs and travel subsidies in Australia could cover essential costs in multiple aspects [32, 33]. However, the continued reporting cost and access barriers among participants suggests that these supports may not be well integrated into survivorship care pathways.

Long wait times for appointments were also frequently reported. These delays can result from system inefficiencies (e.g., insufficient communication or coordination), gaps in healthcare provider knowledge or role clarity, and patient-related factors such as preference for cancer specialists for follow-up care and limited awareness of survivorship needs [8]. These barriers hinder patients’ transition to normal life post-cancer and indicate the importance of reducing delays to improve the quality of life.

One of the most effective solutions for reducing long wait times in cancer survivorship care is the implementation of shared-care models [34]. These models involve coordinated follow-up care between oncology specialists and primary care providers (PCPs), allowing responsibilities to be distributed more evenly across the healthcare system. This approach reduces pressure on specialist services, improves access to timely follow-up, and enhances continuity of care by monitoring for recurrence, managing comorbidities, and addressing psychosocial needs.

A notable finding in this study is the association between educational level and effectiveness of care after treatment. Cancer survivors with higher educational levels were significantly less likely to rate their care as effective. This may reflect that individuals with higher levels of education tend to have higher expectations for healthcare services and greater awareness of gaps in their care. More educated individuals are often more proactive in seeking specialised support, and when confronted with systemic issues such as long wait times or poor coordination, they may be more critical of the care received [35].

Finally, gender disparities were also observed, with women more likely to report difficulties in accessing supportive care services. This issue is particularly significant among breast cancer survivors. A qualitative study revealed that many experienced psychological distress and changes in quality of life, highlighting the need for improved supportive care services [36]. Additionally, structural sexism within the healthcare system may contribute to these disparities, as female patients often face underfunding for cancers that predominantly affect women, and lower enrolment rates in clinical trials [37]. Addressing these inequities requires targeted policy and system-level reforms to ensure that all survivors receive equitable access to high-quality and comprehensive survivorship care.

## Practical implications

The findings from this study highlight several key areas for improvement in survivorship care delivery in New South Wales:

1. **Improving information dissemination:** Ensuring that all cancer survivors receive clear, accessible information about available supportive care services. Standardised protocols for discussing survivorship care at the end of treatment could help close this gap. These protocols could be disseminated through a combination of clinical education, digital communication channels, and integration into routine practice across health services.
2. **Expanding SCPs:** SCPs were strongly associated with better patient experiences in this study. Including SCPs as a standard component of the end-of-treatment process and integrating them into electronic health records could help enhance uptake and consistency. Implementation may require a coordinated, statewide policy directive supported by investment in health IT infrastructure, as well as training for clinical staff. Whether this change is driven at the state level or hospital level remains a consideration for health system planners.
3. **Addressing financial barriers:** Cost was frequently mentioned as a challenge to accessing supportive care. Policies that subsidise or fully cover essential services, such as psychological support and rehabilitation, as well as financial assistance programs and telehealth services could help ease the burden and improve access for cancer survivors.
4. **Enhancing care coordination:** Survivors often navigate between multiple healthcare providers. Improving communication between specialists and GPs, and additional training for primary care providers in survivorship care management, could help address continuity-of-care issues.

## Limitations and future directions

While this study provides meaningful insights, it has several limitations. First, the relatively small sample size may have limited statistical power and contributed to unstable regression estimates, inflated standard errors, and wide confidence intervals for some variables. Second, recruitment from Cancer Council NSW’s CanAct Community may have introduced selection bias, as participants may be more engaged or informed than the general survivor population, potentially underrepresenting survivors experiencing greater barriers to healthcare access. Third, limited engagement with CALD and Aboriginal and Torres Strait Islander communities, and overrepresentation of breast cancer participants (39.3%) and female participants (72.2%) restricted the applicability of the findings to other cancer types and genders. In addition, the survey items were not independently validated against objective measures, which may affect the accuracy or generalisability of perceived support needs and service effectiveness [38]. The online-only format may have excluded individuals less comfortable with digital platforms. The reliance on self-reported data introduces potential recall bias, especially for sensitive topics such as financial or health-related experiences [39]. Additionally, factors such as patient-provider communication quality and individual health literacy were not assessed, which could provide further context for the observed trends. Furthermore, although the use of complete case analysis reduced statistical power due to the exclusion of incomplete observations, Little’s chi-squared test [40] provided no evidence that the missing data mechanism was not Missing Completely at Random (MCAR). Consequently, substantial bias in the estimated associations arising from the use of complete case analysis was considered unlikely. Several odds ratios were unusually extreme, and some confidence intervals were wide, indicating imprecision in both the point and precision estimates. Although events-per-variable ratios were generally adequate, these findings may reflect quasi-complete separation [41] within some covariate categories, which can result in unstable logistic regression estimates.

Future research should include larger and more diverse survivor populations, particularly among CALD communities, Aboriginal and Torres Strait Islander peoples, and underrepresented cancer groups. Qualitative methods, such as interviews with survivors and healthcare providers, may also provide deeper insight into survivors’ experiences navigating supportive care services and the underlying causes of unmet needs. Further evaluation of survivorship care planning, care coordination, and supportive care accessibility across different healthcare settings may help inform strategies to improve survivorship care delivery and reduce unmet supportive care needs among cancer survivors in NSW.

## Conclusion

This study explored key barriers in cancer survivorship care in NSW, particularly around information access, care coordination, financial burden, and support for certain groups, including women and those without a SCP. Significant gaps in available supportive care information, accessing supportive care services, and uptake of SCPs highlight the need for more consistent survivorship information, improved access to supportive care services, and greater implementation of survivorship care plans in NSW.

## Supplementary Information

Below is the link to the electronic supplementary material.

Supplementary Material 1 (PDF 188 KB)

Supplementary Material 2 (DOCX 84.9 KB)

## Data Availability

No datasets were generated or analysed during the current study.

## Appendix 1

**Table 1:** Association between effectiveness of care after treatment and potential predictors

Factors	Crude OR (95% CI)	*P* value	Adjusted OR (95% CI)	*P* value
Age	0.95 (0.91, 0.99)	0.016**	0.94 (0.88, 1.01)	0.079
Gender Woman Man Non-binary	Reference 0.41 (0.15, 1.08) 6.15 (0.52, 143.05)	0.0736* 0.160	1.03 (0.30, 3.62) 2.67 (0.21, 65.27)	0.959 0.462
Aboriginal and Torres Strait Islander Yes No	3.26 (0.44, 28.85) Reference	0.245	–	–
Country of birth Australia Other	Reference 1.17 (0.38, 3.58)	0.779	–	–
Place of residence Metropolitan Regional	Reference 1.24 (0.54, 2.84)	0.612	–	–
Educational level Primary/secondary school Certificate or diploma Undergraduate degree Postgraduate degree	Reference 13.50 (2.53, 87.85) 8.72 (1.48, 61.28) 11.91 (2.31, 75.27)	0.003** 0.020** 0.004**	18.67 (3.21, 132.54) 7.33 (1.09, 57.93) 12.26 (2.07, 87.61)	0.002** 0.046* 0.008**
Employment status Not retired Retired	Reference 0.44 (0.18, 1.03)	0.0621*	1.29 (0.38, 4.48)	0.683
Type of cancer Breast cancer Prostate cancer Bowel cancer Melanoma/skin cancer Other	Reference 0.47 (0.13, 1.59) 0.63 (0.14, 2.85) 0.15 (0.02, 0.90) 1.35 (0.41, 4.51)	0.227 0.550 0.040 0.625	– – – –	– – – –
Stage of cancer Stage 0 Stage 1 Stages 2 and 3 Stage 4	Reference 1.65 (0.39, 7.20) 3.26 (0.83, 13.25) 2.15 (0.37, 12.96)	0.501 0.091 0.396	– – –	– – –
Whether received written or digital survivorship care plan Yes No	Reference 7.54 (2.07, 30.74)	0.003**	12.80 (0.21, 65.27)	< 0.001***

a. *OR* odds ratio, *CI* confidence interval

b. *Significant at *P* < 0.1; **significant at *P* < 0.05; ***significant at *P* < 0.001

c. Age and gender were forced into the multivariable model. Predictors (residence and type of cancer) retained in the modelling if *P* < 0.1 in the univariable

**Table 2:** Association between easiness to access supportive care services and potential predictors

Factors	Crude OR (95% CI)	*P* value	Adjusted OR (95% CI)	*P* value
Age	0.98 (0.94, 1.03)	0.461	1.03 (0.98, 1.09)	0.234
Gender Woman Man Non-binary	Reference 0.09 (0.02, 0.28) 4.18 (0.31, 101.42)	< 0.001 0.284	0.08 (0.01, 0.42) 5.58 (0.37, 144.75)	0.004** 0.217
Aboriginal and Torres Strait Islander Yes No	2.02 (0.24, 19.54) Reference	0.513	–	–
Country of birth Australia Other	Reference 0.66 (0.21, 2.03)	0.466	–	–
Place of residence Metropolitan Regional	Reference 1.09 (0.47, 2.53)	0.838	–	–
Educational level Primary/secondary school Certificate or diploma Undergraduate degree Post-graduate degree	Reference 2.39 (0.51, 11.39) 1.80 (0.36, 9.06) 2.47 (0.55, 11.31)	0.267 0.707 0.235	– – –	– – –
Employment status Not retired Retired	Reference 0.75 (0.32, 1.74)	0.499	–	–
Type of cancer Breast cancer Prostate cancer Bowel cancer Melanoma/skin cancer Other	Reference 0.11 (0.02, 0.45) 0.29 (0.07, 1.13) 0.28 (0.04, 1.84) 0.48 (0.14, 1.60)	0.003** 0.075* 0.188 0.234	0.93 (0.11, 8.11) 0.73 (0.16, 3.43) 0.67 (0.09, 4.58) 1.15 (0.31, 4.29)	0.950 0.686 0.680 0.834
Stage of cancer Stage 0 Stage 1 Stages 2 and 3 Stage 4	Reference 0.92 (0.19, 4.51) 2.91 (0.62, 13.76) 3.13 (0.49, 20.36)	0.923 0.172 0.226	– – –	– – –
Whether received written or digital survivorship care plan Yes No	0.19 (0.05, 0.66) Reference	0.010**	0.37 (0.08, 1.55)	0.178

a. *OR* odds ratio, *CI* confidence interval

b. *Significant at *P* < 0.1; **significant at *P* < 0.05; ***significant at *P* < 0.001

c. Age and gender were forced into the multivariable model. Predictors (residence and type of cancer) retained in the modelling if *P* < 0.1 in the univariable
