# Supplementary material for: Exploring the barriers to optimal survivorship care for people living with Cancer in NSW
Source: Support Care Cancer. 2026 Jul 1;34(7):711. doi: 10.1007/s00520-026-10921-6 (PMC13319190; doi:10.1007/s00520-026-10921-6)
Supplement: Supplementary file 1 — Supplementary Material 1 (PDF 188 KB) [file 520_2026_10921_MOESM1_ESM.pdf]

EXPLORING THE BARRIERS TO OPTIMAL SURVIVORSHIP CARE FOR PEOPLE LIVING WITH CANCER IN NSW

Authors: Yuqi Ouyang[1], Helen Tran[1], Brad Gellert[1], Elizabeth Kennedy[2], Dr. Carolyn Mazariego-Jones[2], Professor Michael David[3, 4], Professor Janette Vardy[5, 6]

1. Policy and Advocacy Unit, Cancer Council NSW, NSW, Australia
2. School of Population Health, UNSW, NSW, Australia
3. The Daffodil Centre, University of Sydney, a joint venture with Cancer Council NSW, Sydney, Australia
4. School of Medicine & Dentistry, Griffith University, QLD, Australia
5. Sydney Cancer Survivorship Centre, Concord Hospital, NSW, Australia
6. Faculty of Medicine and Health, University of Sydney, NSW, Australia

Corresponding Author: Dr Janette Vardy BMed (Hons), PhD, FRACP

Professor of Cancer Medicine

University of Sydney

Medical Oncologist

Director of Sydney Cancer Survivorship Centre

Concord Cancer Centre, Concord Repatriation & General Hospital

Hospital Rd, Concord, NSW, 2139, Australia

+612 9767 5000

janette.vardy@sydney.edu.au

Table S1 Characteristics of patients diagnosed with cancer from Cancer Council NSW's Survivor Survey 2023 (n = 209).

Factors	n	Relative frequency, %
Age, years		
< 60	51	24.6
60 – 65	50	24.2
66 – 74	54	26.1
> 74	52	25.1
Gender		
Woman	151	72.2
Man	55	26.3
Non-binary	3	1.4
Aboriginal and Torres Strait Islander		
Yes	4	2.0
No	201	98.0
Place of residence		
Metropolitan	113	54.6
Regional	94	45.4
Country of birth		
Australia	166	79.8
Other	42	20.2
Educational level		
Primary or Secondary school	25	12.2
Certificate or Diploma	63	30.7
Undergraduate degree	56	27.3
Postgraduate degree	61	29.8
Employment status		
Retired	99	48.5
Not retired	105	51.5
Type of cancer		
Breast cancer	81	39.3
Bowel cancer	18	8.7
Melanoma/skin cancer	17	8.3
Prostate cancer	29	14.1
Other	61	29.6
Stage of cancer		
Stage 0	28	16.6
Stage 1	38	22.5
Stage 2 & 3	87	51.5
Stage 4	16	9.5
Whether received written or digital survivorship care plan		
Yes	20	10.9
No	164	89.1
Whether received information about supportive services		
Yes	124	64.2
No	69	35.8
Easiness to access supportive care services		
Very easy	21	15.4
Easy	40	29.4
A little difficult	55	40.4
Very difficult	20	14.7
Whether support met patients' needs		
Yes	114	72.2
No	44	27.8

a. Missing values for age, Aboriginal and Torres Strait Islander, country of birth, place of residence, educational level, employment status, type of cancer, stage of cancer, receipt of survivorship care plan, receipt of information about supportive services, and whether support met patients' needs were 2, 4, 1, 2, 4, 5, 3, 40, 25, 16, 51, respectively.

Table S2 Association between whether received supportive care services and potential predictors.

Factors	Crude OR (95% CI)	P value	Adjusted OR (95% CI)	P value
Age	1.02 (0.97, 1.08)	0.376	1.03 (0.97, 1.09)	0.314
Gender				
Woman	Reference			
Man	0.85 (0.28, 2.68)	0.769	0.63 (0.17, 2.27)	0.472
Non-binary	0.44 (0.03, 5.72)	0.496	0.61 (0.04, 8.30)	0.683
Aboriginal and Torres Strait Islander				
Yes	0.77 (0.10, 8.81)	0.811	–	–
No	Reference			
Country of birth				
Australia	Reference			
Other	1.17 (0.32, 5.16)	0.818	–	–
Place of residence				
Metropolitan	Reference			
Regional	0.52 (0.19, 1.39)	0.193	–	–
Educational level				
Primary/Secondary school	Reference			
Certificate or diploma	2.83 (0.55, 15.85)	0.211	–	–
Undergraduate degree	3.29 (0.57, 21.22)	0.182	–	–
Postgraduate degree	3.10 (0.62, 16.84)	0.166	–	–
Employment status				
Not retired	Reference			
Retired	0.73 (0.27, 1.95)	0.529	–	–
Type of cancer				
Breast cancer	Reference			
Prostate cancer	0.86 (0.21, 4.08)	0.844	–	–
Bowel cancer	0.40 (0.09, 1.81)	0.231	–	–
Melanoma/skin cancer	0.40 (0.06, 2.90)	0.349	–	–
Other	1.10 (0.28, 5.03)	0.899	–	–
Stage of cancer				
Stage 0	Reference			
Stage 1	2.23 (0.44, 11.59)	0.326	–	–
Stage 2 & 3	3.12 (0.67, 14.87)	0.144	–	–
Stage 4	1.18 (0.20, 6.92)	0.850	–	–
Whether received written or digital survivorship care plan				
Yes	1.48 (0.36, 8.47)	0.603	–	–
No	Reference			

a. OR: odds ratio; CI: confidence interval

b. *Significant at $P < 0.1$; **Significant at $P < 0.05$; ***Significant at $P < 0.001$.

c. Age and Gender were forced into the multivariable model. Predictors (residence and type of cancer) retained in the modelling if $p < 0.1$ in the univariable.

Table S3 Association between whether support met patients' needs and potential predictors.

**Factors**	**Crude OR (95% CI)**	**P value**	**Adjusted OR (95% CI)**	**P value**
Age	1.02 (0.97, 1.07)	0.523	1.00 (0.94, 1.06)	0.892
Gender				
Woman	Reference			
Man	1.42 (0.45, 5.22)	0.564	1.48 (0.40, 6.16)	0.563
Non-binary	0.09 (0.00, 1.16)	0.066*	0.09 (0,00, 1.22)	0.071
Aboriginal and Torres Strait Islander				
Yes	0.05 (0.00, 0.59)	0.015**		
No	Reference			
Country of birth				
Australia	Reference			
Other	1.83 (0.46, 10.25)	0.406	–	–
Place of residence				
Metropolitan	Reference			
Regional	0.98 (0.37, 2.66)	0.971	–	–
Educational level				
Primary/Secondary school	Reference			
Certificate or diploma	1.00 (0.15, 5.37)	1.000	–	–
Undergraduate degree	0.87 (0.12, 5.18)	0.878	–	–
Postgraduate degree	1.10 (0.17, 5.69)	0.916	–	–
Employment status				
Not retired	Reference			
Retired	0.84 (0.31, 2.28)	0.728	–	–
Type of cancer				
Breast cancer	Reference			
Prostate cancer	1.09 (0.27, 5.10)	0.904	–	–
Bowel cancer	2.55 (0.48, 25.95)	0.293	–	–
Melanoma/skin cancer	4.58 (0.44, 624.53)	0.237	–	–
Other	0.69 (0.19, 2.60)	0.579	–	–
Stage of cancer				
Stage 0	Reference			
Stage 1	1.08 (0.16, 6.03)	0.928	–	–
Stage 2 & 3	0.90 (0.15, 4.26)	0.896	–	–
Stage 4	0.45 (0.06, 2.75)	0.395	–	–
Whether received written or digital survivorship care plan				
Yes	1.38 (0.33, 7.90)	0.671	–	–
No	Reference			

a. OR: odds ratio; CI: confidence interval

b. *Significant at P < 0.1; **Significant at P < 0.05; ***Significant at P < 0.001.

c. Age and Gender were forced into the multivariable model. Predictors (residence and type of cancer) retained in the modelling if p < 0.1 in the univariable.
