# Supplementary material for: Exploring the barriers to optimal survivorship care for people living with Cancer in NSW
Source: Support Care Cancer. 2026 Jul 1;34(7):711. doi: 10.1007/s00520-026-10921-6 (PMC13319190; doi:10.1007/s00520-026-10921-6)
Supplement: Supplementary file 2 — Supplementary Material 2 (DOCX 84.9 KB) [file 520_2026_10921_MOESM2_ESM.docx]

**Appendix 2**

**Research Protocol, Version 5, 21 August 2024**

21 August 2024

**Policy and Advocacy; Cancer Prevention and Advocacy Division**

Contents

[1. Protocol Synopsis 4](#_Toc175131942)

[2. Study Management 5](#_Toc175131943)

[2.1. Project Team Roles & contribution 5](#_Toc175131944)

[2.2. Funding and Resources 5](#_Toc175131945)

[2.3. Financial Disclosure and Conflicts of Interest 6](#_Toc175131946)

[2.4. Proposed Research Timeline 6](#_Toc175131947)

[3. Introduction 6](#_Toc175131948)

[3.1. Background and Rationale 6](#_Toc175131949)

[3.1.1. What is optimal survivorship care? 6](#_Toc175131950)

[3.1.2. What does the current literature say about provision of survivorship care and gaps/ system barriers in Australia? 8](#_Toc175131951)

[3.1.3. What gap will this current study aim to address? 8](#_Toc175131952)

[3.2. Research Aims and Objectives 8](#_Toc175131953)

[3.2.1. Are cancer survivors receiving optimal cancer survivorship care? 8](#_Toc175131954)

[Methods 9](#_Toc175131955)

[3.3. Study Design 9](#_Toc175131956)

[3.4. Participating Sites 9](#_Toc175131957)

[3.5. Project Duration 9](#_Toc175131958)

[3.6. Data Collection 9](#_Toc175131959)

[3.7. Sample Size 9](#_Toc175131960)

[3.8. Eligibility Criteria 10](#_Toc175131961)

[3.8.1. Cancer Survivors 10](#_Toc175131962)

[3.9. Participant Recruitment and Consent Procedure 10](#_Toc175131963)

[3.9.1. Recruitment and Consent Procedure 10](#_Toc175131964)

[3.10. Participant Withdrawal 10](#_Toc175131965)

[3.11. Adverse Event Reporting 11](#_Toc175131966)

[4. Data Management 12](#_Toc175131967)

[4.1. Data Storage and Retention 12](#_Toc175131968)

[4.2. Data Analysis 13](#_Toc175131969)

[4.2.1. Quantitative Data (Surveys) 13](#_Toc175131970)

[4.3. Data Confidentiality 13](#_Toc175131971)

[5. Results 14](#_Toc175131972)

[5.1. Provision of Results to Participants 14](#_Toc175131973)

[5.2. Dissemination and Publication 14](#_Toc175131974)

[5.3. Amendments 14](#_Toc175131975)

[5.4. Project Closure 14](#_Toc175131976)

[6. References 15](#_Toc175131977)

1. **Protocol Synopsis**

| **Title** |  |
| --- | --- |
| **Background** | With cancer incidence expected to rise significantly over the next 25 years, optimal survivorship care is now more important than ever to meet the ongoing needs of cancer survivors throughout the cancer care continuum.  Considerable variations in the provision of survivorship care exist across the health system in Australia. A 2018 survey of 483 patients with colorectal cancer in NSW revealed that only 23% of patients received a follow-up care plan and less than half of participants received guidelines recommended follow-up care (Young et al.). Several studies have identified barriers to optimal survivorship care, including healthcare workforce, resourcing issues, and lack of health professional reimbursement for survivorship care (Halpern et al.) This present study aims to build on this evidence by exploring the provision of optimal survivorship care in NSW and potential system barriers. |
| **Research Objectives** | This research aims to understand the extent of optimal survivorship care received by cancer survivors in NSW. It also aims to understand the system barriers to optimal survivorship care to inform government policy recommendations. |
| **Research Design** | The study will use a quantitative methodology consisting of a written survey of cancer survivors via the CanAct community (CCNSW). The named research group will carry out all research activities in this protocol and be responsible for all project elements, including data collection, security, analysis, and write-up. Participants will be purposively sampled to ensure an even distribution of participation in groups can be achieved and data saturation is feasible. |
| **Sample Size** | Survey participants (cancer survivors) *n=400* |
| **Selection Criteria and Recruitment** | Cancer survivors, including survivors with current and past cancer diagnoses, will be recruited via the CanAct community, Cancer Council NSW social media and online mailing lists, and through networks and stakeholders (e.g. Cancer Voices NSW). Ekas Market Research Services Australia will also assist in promotion by sending a link to the survey to their mailing lists, which includes people who have indicated that they want to be sent this type of opportunity. An incentive will be offered, where respondents will go into the draw to win a $100 Woolworths e-gift card. |
| **Consent process** | Completion and return of the participant information statement/consent form will be taken as consent to participate in the project. All forms will be held electronically via Cancer Council NSW secure servers (see below for data management process). |
| **Data Management** | In line with established CCNSW processes and procedures and as governed by The University of Sydney Human Ethics Approval (HREC Ref: 2022/769).  Cancer Council NSW electronic research data may also be stored 1) on a secure local network whose servers are located in Cancer Council NSW, a swipe card access restricted building, and 2) on Microsoft SharePoint, a secure web-based application for document management and storage. To ensure the highest security, Cancer Council NSW's data housed on web-based platforms are stored exclusively in datacentres in Australia (New South Wales and Victoria) and comply with Australia's stringent privacy standards (ISO 27018).  Only research personnel can access the research data via secure logins and password-protected computers. The data are managed in accordance with the Australian Code for the Responsible Conduct of Research and Cancer Council NSW. Further information about data management is available in section 5 of this protocol. |

1. **Study Management**
   1. **Project Team Roles & contribution**

| **Name** | **Position** | **Contribution** | **Expertise** |
| --- | --- | --- | --- |
| **Professor Janette Vardy** | Professor of Cancer Medicine, University of Sydney  Medical Oncologist, Concord Cancer Centre  Director of Sydney Cancer Survivorship Centre | Overall oversight of the project design, methods, conduct, write up; support for translation and dissemination activities | Psycho-oncology and survivorship field expert |
| **Jessica Sheppard** | Health Programs Lead, Medibank | Overall oversight of the project design, methods, conduct, write up; support for translation and dissemination activities | Policy expertise |
| **Helen Tran** | Cancer Policy Lead, Policy and Advocacy Unit; Cancer Prevention and Advocacy Division | Recruitment of participants, analysis, and write up | Policy expertise |
| **Elizabeth Kennedy** | Research Officer, School of Population Health, UNSW | Support data collection and advice on survey design. | Cancer research |
| **Brad Gellert** | Manager, Policy and Advocacy Unit; Cancer Prevention and Advocacy Division | Advice on policy-relevant design; aid in translation and dissemination | Policy expertise |
| **Martha Gerges** | Project Development Manager, The George Institute for Global Health | Project coordination and execution of the design and methods. Support analysis and write up. | Cancer research |
| **Dr Carolyn Mazariego-Jones** | Senior Research Fellow, School of Population Health, UNSW | Project design, methodological advice, write up; support for translation and dissemination | Epidemiology, Psycho-oncology & Behavioural Researcher |
| **Professor Michael David** | Stream Led, Research Methods, The Daffodil Centre | Support analysis and write up. | Research methods and statistics |
| **Yuqi Ouyang** | Research Volunteer, Cancer Council NSW | Support analysis and write up. | Research in public health |

- 1. **Funding and Resources**

This project is funded and resourced within the internal Policy and Advocacy Team, Cancer Prevention and Advocacy Division at the Cancer Council NSW. The resources required for this research project include the investment of time by Cancer Council NSW and the Daffodil Centre staff to develop study materials, collect and clean the data, analyse data, interpret results, and disseminate findings.

- 1. **Financial Disclosure and Conflicts of Interest**

There are no conflicts of interest for the project. The $100 Woolworths e-gift card will be used as an incentive to recruit survey participants. The e-gift card is funded within the internal Policy & Advocacy Unit, Cancer Prevention and Advocacy Division at Cancer Council NSW. The Project Team will not offer incentives to external organisations for assistance or participation in this research. Ekas Market Research Services Australia will assist on a pro bono basis.

- 1. **Proposed Research Timeline**

| **Activity** | **Date** |
| --- | --- |
| Research design and scoping phase | July-August 2022 |
| HREC review and approval | December 2022 |
| Survey completions and data collection | February-December 2023 |
| Data review and analysis | January-December 2024 |
| Research outputs (e.g., internal reports, publications) | From January 2025 |

1. **Introduction**
   1. **Background and Rationale**

Optimal survivorship care is important to ensure the health and wellbeing of cancer survivors from the point of diagnosis through to the end of active treatment. With cancer incidence expected to significantly rise over the next 25 years [41], optimal survivorship care is now more important than ever to meet the ongoing needs of cancer survivors throughout the cancer care continuum.

Traditionally survivorship care has focused on monitoring for disease surveillance and recurrence, however, it has become apparent that cancer survivors experience a number of gaps in post- treatment care including inadequate support for physical and emotional needs as well as poor communication and coordination amongst their health care teams [4].

While people can live well after cancer treatment, many experience a broad range of physical, psychosocial, and financial issues that impact their quality of life [42]. These issues burden individuals living with cancer, their families and carers, and the broader community. Thus, a huge pressure is placed on the health system to ensure it is able to meet the growing cancer survivor population [43, 44].

- - 1. **What is optimal survivorship care?**

In Australia, there are a number of survivorship frameworks that currently exist based on recurring recommendations that effective systems should be based on a multidisciplinary approach that factors in individual needs (assessment based). Cancer Australia's Principles of Cancer Survivorship [45] outlines five principles that drive cancer survivorship care including consumer involvement and person-centred care, support for living well, evidence-based pathways, coordinated and integrated care and data-driven improvements and investment in research [45]. In addition, Cancer Australia outlines six categories or domains of supportive care needs for cancer survivors in Australia (see table below). Through these guiding principles, we can identify what optimal survivorship care covers and the domains that need to be considered [45].

Moreover, the Clinical Oncology Society of Australia's (COSA) Model of Survivorship Care outlines guiding principles for quality survivorship care in Australia including key recommendations based on different stages of the cancer care continuum (e.g., diagnosis, end of primary treatment, follow-up care) [46].

According to COSA's model, the five fundamental princples underpinning optimal survivorship care are:

- Suvivor centred; enabling decision-makers to put survivors at the forefront of all decisions and models
- Coordinatored across all services
- Integrated across all levels of service delivery at all time points throughout the cancer continuum
- Accessible and equitable to all people
- Promote overall wellbeing, prevent future illness and effectively engage to manage problems and symptoms.

In addition to the national frameworks and policies, some states and territories in Australia have also implemented state-based guidelines for the management of survivorship care including:

- South Australian Statewide Survivorship Framework
- Victorian Cancer Survivorship Program
- Western Australian Cancer Plan with specific focus on survivorship.

Thus, for care to be opitmal, it needs to factor in overall health and wellbeing, rather than focusing on the cancer itself. Survivorship care needs to focus on living beyond cancer through a highly integrated and dynamic system that can meet the needs of the population. This includes addressing needs that may be physical, psychological, social, cultural, informational or cultural as identified by Cancer Australia [45]. While highly interconnected, each domain outlines sub-areas that it covers [45].

| **Supportive Care Need Domain [45]** | **Definition** |
| --- | --- |
| Physical | A wide range of physical symptoms that cancer survivors face for months or years after treatment completion. These include short- and long-term physical impacts of a cancer diagnosis and treatment and may require acute or ongoing intervention (e.g., weight loss, pain, tiredness, shortness of breath) [47, 48]. |
| Psychological | Issues relating to an individual's mental well-being and personal relationships (e.g., anxiety, fear of cancer recurrence, anger, depression, stress) [47, 49]. |
| Social and Cultural | An individual's ability to hold and maintain relationships with others, including emotional support and financial concerns (e.g., acculturation, morals, attitudes, and beliefs) [47, 49]. |
| Information | Information related to the above sections concerning an individual's ability to access and use relevant and reliable health information about their disease, treatment and support services available [47, 48]. |
| Spiritual | An individual's changing sense of self due to their cancer diagnosis and treatment. This domain deals with underlying beliefs and concerns regarding aspects such as feelings of guilt or shame [47, 48]. |

- - 1. **What does the current literature say about provision of survivorship care and gaps/ system barriers in Australia?**

The cancer care paradigm must shift to recognise the survivorship care needs of Australian cancer patients and their families better in line with the needs of its population. The current literature highlights the complexity of the Australian health care system that leaves patients and their loved ones feeling overwhelmed and confused, at an already emotionally-charged time [50]. Many studies have highlighted affordability and funding as the greatest barrier to implementing effective/optimal survivorship care [51-53] and building stronger and more evidence-based business models as the solution [51, 52]. In addition, cultural awareness of the importance of survivorship care in Australia needs to continue to evolve. A recent survey of Australian cancer care providers found that the most critical service gap was services that aim to meet the broader biopsychosocial and long-term care needs of cancer survivors [49].

Moreso, in a national survey conducted in 2019, Hunter and her colleagues found that the range of service provisions and the variations between service delivery in Australia highlighted the complexity of the system and the system's challenge to meet the broader bio-psycho-social needs of cancer patients [50]. Furthermore, the lack of integrated services that offered support to survivors across more than two domains was highlighted, promoting the need for public and private sectors to change their models of working [45, 50, 54].

Moreover, the identification of a service gap relating to the need for more specialist oncology services within remote/rural regions of Australia is emphasised [50, 55]. Moreover, in a recent study exploring the barriers and facilitators to integrative oncology services in Australia, authors identified that gaps are substantial and there were no integrative oncology services in many regional/remote parts of Australia [51].

Cancer supportive care needs are frequently discussed in identifying gaps in cancer survivors or unmet needs. A plethora of work has been undertaken in identifying unmet needs, but **little attention to policy solutions to address these needs at a system level**. Previous reviews have focused on identifying the unmet needs of patients within the cancer care continuum and the implications for clinical practice; however, none have focused on policy considerations and real-life translation from evidence to practice [1, 5], identifying a huge gap in the provision of survivorship care in Australia.

- - 1. **What gap will this current study aim to address?**

The proposed research aims to build on the existing literature by exploring the provision of optimal survivorship care and understanding the system barriers from an NSW perspective. Findings from this work can help identify policy solutions to improving survivorship care across the state that address the individual needs of cancer survivors.

- 1. **Research Aims and Objectives**

The aim of this research is to understand the extent of optimal survivorship care received by cancer survivors in NSW. It also aims to understand the system barriers to optimal survivorship care to inform policy recommendations to government. This research aims to address two key research questions:

- - 1. **Are cancer survivors receiving optimal cancer survivorship care?**

We will explore whether cancer survivors have received optimal survivorship care by determining whether:

- Cancer survivors received a treatment summary and survivorship care plan including diagnosis, treatment and follow up care.
- A needs assessment was conducted by the health professional to determine social, emotional, occupational, educational, lifestyle and spiritual needs.
- Cancer survivors received adequate information on cancer survivorship according to the Institute of Medicine essential components of survivorship care.
  - 1. **What are the system barriers and enablers to optimal survivorship care in NSW?**

A secondary aim of this research is to explore the system barriers to achieving optimal survivorship care. This will inform potential government policies in this area and support our election asks.

**Methods**

- 1. **Study Design**

The study will utilise a quantitative research methodology consisting of a written survey. The named research group will carry out all research activities in this protocol. Participants will be purposively sampled to ensure an even distribution of group participation can be achieved and data saturation is feasible.

- 1. **Participating Sites**

The project team will manage all research activities at Cancer Council NSW. The research aims to capture cancer survivors' experiences with survivorship care and due to this, recruitment methods identified for this project will not require Specific Site Assessment governance at individual cancer treatment sites/locations.

- 1. **Project Duration**

It is estimated that the project will run for 7 months depending on the ability to reach data saturation, prepare and present dissemination and other project factors. Data collection will be open until data saturation has been reached. Primary data analysis will follow recruitment closure and will encompass a thematic analysis of the qualitative content.

- 1. **Data Collection**

Data collected throughout the study will be restricted to the project team, and all data will be stored within Australia to meet ethical requirements. All written survey responses will be stored on secure CCNSW servers that align with required legislative guidelines in Australia. All data will be stored within the Cancer Council NSW secure servers and kept for a minimum standard, five years, as required by law (see Section 5 for further detail).

- 1. **Sample Size**

The non-probability sampling technique of convenience sampling will be utilised. Due to the large population group who can identify as cancer survivors in NSW and research limitations, it is not feasible to choose a realistic sample that is representative of the population [56]. Therefore, the research team, utilising their expertise in this field aim to recruit 400 cancer survivors.

- 1. **Eligibility Criteria**
     1. **Cancer Survivors**

Cancer survivors (n=400) will be invited to participate in this research project. Cancer survivors must satisfy the following specific criteria:

- Aged over 18 years
- Ability to provide informed consent (self-rated)
- Be in any stage of the cancer continuum, including:
  - Recently diagnosed
  - Undergoing active treatment
  - In remission/past diagnosis.

Patients identified as being in palliative care will be excluded for the purposes of this research in order to be respectful and sensitive to their current situation.

- 1. **Participant Recruitment and Consent Procedure**
     1. **Recruitment and Consent Procedure**

Passive snowball sampling will be utilised as a recruitment method during the research project. This will allow participants or potential participants to promote the research project by providing information about the research (e.g., advertisement, link to the project PIS, or invitation email) to people they may know. This will allow the project team to potentially identify suitable participants throughout the recruitment process [15]. All potential participants will be screened according to the eligibility criteria above.

Specifically, groups will be invited to participate in this project through the following methods:

1. Cancer Council's CanAct Community who will be emailed a direct link to the study documentation via email sent from Cancer Council NSW servers (sourced through CCNSW mailing lists).
2. Cancer Council NSW will create posts using the approved media wording for social media channels and online mailing lists.

The Project Team will send relevant networks and stakeholders (e.g. Cancer Voices NSW) an email with the link to the survey for distribution to clients and/or members. Also, Ekas Market Research Services Australia will assist in promotion by sending a link to the survey to their mailing lists, which includes people who have indicated that they want to be sent this type of opportunity. An incentive will be offered to recruit survey participants. Respondents will go into the draw to win a $100 Woolworths e-gift card. Interested participants will be directed to an online form containing the participant information statement and demographics, and consent form through all avenues above. Participants will have the opportunity to ask any questions and be given the contact information of the research team as appropriate. They will also be reminded that choosing not to participate will have no impact on their relationship with any affiliated person or organisation.

Participants will be prompted to read the patient information statement and read and complete the consent form via the online form. All eligibility questions will be asked through this form and informed consent obtained. Details to contact the research team will also be included.

Participants will also be asked basic demographic questions attached to the consent form during study sign-up online. Screening and eligibility will be confirmed at the beginning of the interview with a research team member.

- 1. **Participant Withdrawal**

Participation in the study is voluntary. Consenting participants may withdraw at any time until they complete the study interview, without giving a reason. Withdrawal will not affect their relationship with Cancer Council NSW or the assistance they receive from Cancer Council NSW now or in the future. If participants choose to end the interview early, they will be asked whether the existing data collected may be used for the research and their response recorded for data management purposes. If the participant does not wish their data to be used, recordings will be deleted, and data excluded. Participants will be advised in the PIS that data is re-identifiable.

- 1. **Adverse Event Reporting**

It is anticipated that there is a low risk of harm to individuals participating in this research. The contact details of the research team and HREC will be provided in the participant information statement, in addition to the 13 11 20 information and support number and other relevant helplines should they require any support.

All identifiable data will be removed from interview transcripts to ensure that participants' privacy and confidentiality are respected. This involves assigning a pseudonym to participants and removing the names of family members, medical staff, and treatment locations. All data will be stored in re-identifiable form, meaning identifying information such as name and contact details will be removed from participant data. Participants will be assigned a unique alphanumeric ID, which will be used to identify data such as electronic audio files of participant interviews.

1. **Data Management**
   1. **Data Storage and Retention**

The data will be managed in accordance with the Australian Code for the Responsible Conduct of Research and Cancer Council NSW's Information Security Policy, and the following measures will be undertaken:

| Data collection:   1. Consent forms and demographic data 2. Survey responses | Consent forms and all survey responses will be stored separately in secure folders so identifying data cannot be linked.  To ensure the highest security, Cancer Council NSW's data are housed on web-based platforms, stored exclusively in datacentres in Australia (New South Wales and Victoria) and comply with Australia's stringent privacy standards (ISO 27018).  Only the research team will have access to study data. Data will also be deleted in line with the data retention policy specified below. |
| --- | --- |
| Data storage | In addition, Cancer Council NSW electronic research data may also be stored 1) on a secure local network whose servers are located in Cancer Council NSW, a swipe card access restricted building, and 2) in Microsoft SharePoint, a secure web-based application for document management and storage. Only the research team will have access to these data via secure logins and password-protected computers.  In accordance with the Cancer Council NSW's Information Security Policy system, backups of Cancer Council NSW's entire network are stored in secure 3^rd^ party data storage centres in the Sydney area. Management of the system backups is governed by the Information Security Policy, and data storage centres must meet strict security standards.  All paper-based study material and data will be securely stored in locked filing cabinets, tambours or other swipe card accessed areas within the building.  Names and contact details will be collected to conduct the interviews. However, consenting information (names, contact information etc.) will be stored separately to demographic data. Alphanumeric ID codes will be applied and used throughout data collection and reporting so that no single participant can be identified within any research dissemination. |
| Length of time for which research data will be retained | In accordance with the NHMRC's Australian Code for the Responsible Conduct of Research and Management of Data and Information in Research, research data will be kept for five years from the date of last publication, unless other conditions are specified. |
| Data deletion, or destruction | After the data retention period has passed, all research study documentation and data will be destroyed. Paper-based material generated in the conduct of this study will be shredded, and electronic files stored on the local network and secure web-based platforms for document management and storage will be deleted.  The Cancer Council NSW system backups will continue to be stored in secure 3^rd^ party data storage centres in the Sydney area. Management of the system backups is governed by Cancer Council's Information Security Policy and Record Retention Policy. |
| Data transfer with external collaborators | In the case of any future collaborations with additional external researchers, who will be added via an amendment to this approval, the non-identifiable data from this project may be shared for research purposes or other related research. Microsoft SharePoint will be used to facilitate secure file transfer to and data sharing with other external researchers. SharePoint is a secure web-based application for document management and storage which can be accessed by invitation only.  Non-identifiable data may be downloaded onto approved researcher's computers and shared networks for analysis. As part of our agreement with the researchers, data security details at each institution would be obtained prior to sharing the data. The data will be deleted from computers following analysis. |
| Data sharing with external collaborators | If there are requests for data sharing from external collaborators, the recommendations of the NHMRC Open Access policy will be adhered to. Cancer Council NSW will have ongoing custody of the data, and all use of the data will be approved and managed by the Cancer Council NSW. |

- 1. **Data Analysis**
     1. **Quantitative Data (Surveys)**

Descriptive statistics will be used to summarise participant socio-demographic data for both groups. Multivariable logistic regression will be applied to determine different barriers to optimal care between groups while controlling for potential confounders. Univariate analysis will be run based on a 1v1 comparison to identify interactions between variables. Chi-square testing will be undertaken to examine relationships between any categorical variables and correlation testings (Pearson) run for numerical variables.

- 1. **Data Confidentiality**

The data collected includes descriptive and demographic information used to look at different sub-groups within the analysis, for example, age group, gender, and cancer stage. However, the data will remain non-identifiable, as the approved research team will remove names, locations, and other identifying information. Therefore, it will be stored and managed confidentially using the measures outlined above.

To minimise the risk of participant identification, only aggregated data will be used in any publication or distribution of results. Access to the data will be restricted and governed by the Cancer Council NSW research team approved on the project.

1. **Results**
   1. **Provision of Results to Participants**

Information about the provision and dissemination of results will be provided in the participant information sheet. Participants will not be informed of their individual results. Participants will be informed that results based on the aggregated interview data will be written up in a report and/or publication. Should a participant specifically request to view the final report output, this will be shared.

- 1. **Dissemination and Publication**

Results will be presented in a summary report and distributed to interested parties on an as-needed basis. It is planned that the summary of results will be reported internally and externally to appropriate stakeholders, in peer-reviewed publications and presented at scientific conferences. Some findings may also be used for Cancer Council NSW media releases, grant proposals and briefing documents to government agencies, e.g., NSW Health.

- 1. **Amendments**

Any amendments will be submitted to the HREC for review prior to implementation as per HREC guidelines. 

- 1. **Project Closure**

Upon conclusion of the project, a project completion report will be submitted to the Ethics Committee. The dissemination of results via peer-review publications or other channels (e.g., reports to government) may continue after the project's conclusion.

1. **References**

1. Luo, Q., et al., *Cancer incidence and mortality in Australia from 2020 to 2044 and an exploratory analysis of the potential effect of treatment delays during the COVID-19 pandemic: a statistical modelling study.* The Lancet Public Health, 2022. **7**(6): p. e537-e548.

2. Selove, R., et al., *Using Implementation Science to Examine the Impact of Cancer Survivorship Care Plans.* J Clin Oncol, 2016. **34**(32): p. 3834-3837.

3. Lisy, K., et al., *Identifying the most prevalent unmet needs of cancer survivors in Australia: A systematic review.* Asia Pac J Clin Oncol, 2019. **15**(5): p. e68-e78.

4. Butow, P.N., et al., *Psychosocial well-being and supportive care needs of cancer patients living in urban and rural/regional areas: a systematic review.* Support Care Cancer, 2012. **20**(1): p. 1-22.

5. Berman, R., et al., *Supportive Care: An Indispensable Component of Modern Oncology.* Clinical oncology (Royal College of Radiologists (Great Britain)), 2020. **32**(11): p. 781-788.

6. Cancer Australia, *Principles of Cancer Survivorship*. 2017.

7. Vardy, J.L., et al., *Clinical Oncology Society of Australia position statement on cancer survivorship care.* 2019. **48**(12): p. 833-836.

8. Australia, C., *Principles of Cancer Survivorship*, C. Australia, Editor. 2017.

9. Molassiotis, A., et al., *Mapping unmet supportive care needs, quality-of-life perceptions and current symptoms in cancer survivors across the Asia-Pacific region: results from the International STEP Study.* Annals of Oncology, 2017. **28**(10): p. 2552-2558.

10. Sanson-Fisher, R., et al., *The unmet supportive care needs of patients with cancer.* Cancer, 2000. **88**(1): p. 226-237.

11. Hunter, J., et al., *Coverage of cancer services in Australia and providers' views on service gaps: findings from a national cross-sectional survey.* BMC cancer, 2019. **19**(1): p. 1-11.

12. Hunter, J., et al., *Barriers and facilitators to integrative oncology services in Australia: A changed mind set required.* The Journal of Alternative and Complementary Medicine, 2021. **27**(S1): p. S-89-S-98.

13. Halpern, M.T., M.S. McCabe, and M.A. Burg, *The cancer survivorship journey: models of care, disparities, barriers, and future directions.* American Society of Clinical Oncology Educational Book, 2016. **36**: p. 231-239.

14. Dulko, D., et al. *Barriers and facilitators to implementing cancer survivorship care plans*. in *Oncology nursing forum*. 2013. NIH Public Access.

15. Beesley, V.L., C. Alemayehu, and P.M. Webb, *A systematic literature review of trials of survivorship interventions for women with gynaecological cancer and their caregivers.* European journal of cancer care, 2019. **28**(3): p. e13057.

16. Lee, S.J.C., et al., *Care coordination for complex cancer survivors in an integrated safety-net system: a study protocol.* BMC Cancer, 2018. **18**(1): p. 1204.

17. Etikan, I., et al., *Comparison of convenience sampling and purposive sampling.* 2016. **5**(1): p. 1-4.

18. Noy, C., *Sampling Knowledge: The Hermeneutics of Snowball Sampling in Qualitative Research.* International Journal of Social Research Methodology, 2008. **11**(4): p. 327-344.

1. Luo, Q., et al., *Cancer incidence and mortality in Australia from 2020 to 2044 and an exploratory analysis of the potential effect of treatment delays during the COVID-19 pandemic: a statistical modelling study.* The Lancet Public Health, 2022. **7**(6): p. e537-e548.

2. Selove, R., et al., *Using Implementation Science to Examine the Impact of Cancer Survivorship Care Plans.* J Clin Oncol, 2016. **34**(32): p. 3834-3837.

3. Lisy, K., et al., *Identifying the most prevalent unmet needs of cancer survivors in Australia: A systematic review.* Asia Pac J Clin Oncol, 2019. **15**(5): p. e68-e78.

4. Butow, P.N., et al., *Psychosocial well-being and supportive care needs of cancer patients living in urban and rural/regional areas: a systematic review.* Support Care Cancer, 2012. **20**(1): p. 1-22.

5. Berman, R., et al., *Supportive Care: An Indispensable Component of Modern Oncology.* Clinical oncology (Royal College of Radiologists (Great Britain)), 2020. **32**(11): p. 781-788.

6. Cancer Australia, *Principles of Cancer Survivorship*. 2017.

7. Vardy, J.L., et al., *Clinical Oncology Society of Australia position statement on cancer survivorship care.* 2019. **48**(12): p. 833-836.

8. Australia, C., *Principles of Cancer Survivorship*, C. Australia, Editor. 2017.

9. Molassiotis, A., et al., *Mapping unmet supportive care needs, quality-of-life perceptions and current symptoms in cancer survivors across the Asia-Pacific region: results from the International STEP Study.* Annals of Oncology, 2017. **28**(10): p. 2552-2558.

10. Sanson-Fisher, R., et al., *The unmet supportive care needs of patients with cancer.* Cancer, 2000. **88**(1): p. 226-237.

11. Hunter, J., et al., *Coverage of cancer services in Australia and providers' views on service gaps: findings from a national cross-sectional survey.* BMC cancer, 2019. **19**(1): p. 1-11.

12. Hunter, J., et al., *Barriers and facilitators to integrative oncology services in Australia: A changed mind set required.* The Journal of Alternative and Complementary Medicine, 2021. **27**(S1): p. S-89-S-98.

13. Halpern, M.T., M.S. McCabe, and M.A. Burg, *The cancer survivorship journey: models of care, disparities, barriers, and future directions.* American Society of Clinical Oncology Educational Book, 2016. **36**: p. 231-239.

14. Dulko, D., et al. *Barriers and facilitators to implementing cancer survivorship care plans*. in *Oncology nursing forum*. 2013. NIH Public Access.

15. Beesley, V.L., C. Alemayehu, and P.M. Webb, *A systematic literature review of trials of survivorship interventions for women with gynaecological cancer and their caregivers.* European journal of cancer care, 2019. **28**(3): p. e13057.

16. Lee, S.J.C., et al., *Care coordination for complex cancer survivors in an integrated safety-net system: a study protocol.* BMC Cancer, 2018. **18**(1): p. 1204.

17. Etikan, I., et al., *Comparison of convenience sampling and purposive sampling.* 2016. **5**(1): p. 1-4.

18. Noy, C., *Sampling Knowledge: The Hermeneutics of Snowball Sampling in Qualitative Research.* International Journal of Social Research Methodology, 2008. **11**(4): p. 327-344.

**Appendix 3**

External Media Wording

Version 4, 18^th^ April 2

**1. Email**

Cancer Council NSW community members will be invited to learn more about the research project and potentially participate via emails sent from CCNSW’s CanAct leadership team / core team. The team will only approach individuals with which they already have an existing relationship and those who are members of the CanAct Community. In addition, the Project Team will send stakeholder organisations (e.g. Cancer Voices NSW) the email to distribute to clients and/or members. The template for the wording is as follows:

Dear [First Name],

We invite you to participate in a research project conducted by Cancer Council NSW (CCNSW) and supported by The Daffodil Centre. We know that living with and beyond cancer is a very important topic for many of us, so, we’ve designed a research project to understand the provision of care in NSW for people with a current or past cancer diagnosis. This research project aims to identify system improvements in cancer care to create practical solutions that positively impact the lives of those impacted by cancer in NSW.

So, if you or someone you know has been diagnosed with cancer or have had a past diagnosis of cancer, please follow the link below to find out more / take part in this project: <https://bit.ly/41hKXWi>. All responses will be anonymous, and you go into the draw to win a Woolworths e-gift card.

Please be aware that taking part in a research project is entirely voluntary, and you are under no obligation to do so.

Thank you for your time, and please reply to this email if you have any questions.

Kindest Regards

[SIGNATURE BLOCK].

**2. Social Media (OPTIONAL)**

The project will be promoted via Cancer Council NSW’s (CCNSW) Facebook and Instagram pages. These posts will contain minimal information about the project and invite interested individuals to follow a link to find more information. This link will lead to the patient information statement and include contact details for the research team. Only those who agree to participate via this link will be given the opportunity to fill in the consent form (to confirm eligibility and informed consent). The wording of the social media posts are as follows:

- - **Option 1**

Living with and beyond cancer often comes with many challenges. Cancer Council NSW is conducting a research project aiming to improve care for people impacted by cancer in NSW from first diagnosis to follow-up care. So, if you’ve ever been diagnosed with cancer, we need your help in exploring areas to make cancer care better across the state. By taking part, you go into the draw to win a Woolworths e-gift card. If you’re interested, please follow this link to find out more: <https://bit.ly/41hKXWi>

- - **Option 2**

Living with and beyond cancer is essential to getting the most out of life and so, at Cancer Council NSW, we are conducting a research project aiming to improve care for people impacted by cancer in NSW.

If you’ve ever had a cancer diagnosis, we’d like you to take part in this project and share your thoughts on what can be done to improve care across the state. By taking part, you go into the draw to win a Woolworths e-gift card. If you’re interested, please follow this link to find out more: <https://bit.ly/41hKXWi>

- - **Option 3**

Optimal care for people living with and beyond cancer is essential. Cancer Council NSW is conducting a research project which aims to identify improvements in care for people impacted by cancer across the state. If you or someone you know has been diagnosed with cancer or has a past diagnosis of cancer, Cancer Council NSW wants to hear from you. By taking part, you go into the draw to win a Woolworths e-gift card.

If you’re interested, please follow this link to find out more: <https://bit.ly/41hKXWi> and help us shape the future of cancer care in NSW.

**Appendix 4**

Cancer Survivor Survey

Version 1, 8^th^ September 2022

**NOTE TO HREC:**

**This survey is designed to capture the experiences of people living with and beyond cancer i.e., those who have a recent diagnosis and those with a past diagnosis of cancer. For this reason, we will implement SKIP LOGIC so that depending on answers, participants will only see questions relevant to them.**

**For simplicity, we have outlined the full set of questions for the two groups, even though the actual survey online will only be available through one standard link with SKIP LOGIC applied where necessary.**

1. **ALL PARTICIPANTS
   Please begin by telling us a little bit about yourself. Section_1**
2. What is your age? (In years): _______________ s1q1
3. Which of the following best describes your gender? S1q2
   1. Woman
   2. Man
   3. Non-binary
   4. Prefer not to say
   5. Other: _______________ s1q2a
4. Are you of Aboriginal and/or Torres Strait Islander origin? S1q3
   1. No
   2. Yes, Aboriginal
   3. Yes, Torres Strait Islander
   4. Prefer not to say
5. What is your postcode? _______________ s1q4
6. In which country were you born? S1q5
   1. Australia
   2. Prefer not to say
   3. Other (please specify): _______________ s1q5a
7. What is the main language you speak at home? S1q6
   1. English
   2. Mandarin
   3. Arabic
   4. Cantonese
   5. Vietnamese
   6. Italian
   7. Spanish
   8. Prefer not to say
   9. Other (please specify): _______________ s1q6a
8. What is your ancestry / ethnic origin? S1q7
   1. African (e.g., Ghanian, Nigerian, Zimbabwean)
   2. Asian (e.g., Chinese, Japanese, Korean)
   3. Caribbean / Central America (e.g., Cuban, Costa Rican, Puerto Rican)
   4. European (e.g., Croatian, Polish, German)
   5. Oceania (e.g., Australian, New Zealander, Fijian)
   6. Mediterranean (e.g., Greek, Italian, Maltese)
   7. Middle Eastern (e.g., Lebanese, Egyptian, Syrian)
   8. North American (e.g., Canadian, American)
   9. South American (e.g., Brazilian, Argentinian, Venezuelan)
   10. Prefer not to say
   11. Other (please specify): _______________ s1q7a
9. What is the highest level of education you have completed? S1q8
   1. No schooling completed
   2. Primary school
   3. Secondary school – high school?
   4. Certificate or diploma
   5. University degree
   6. Post-graduate degree
   7. Prefer not to say
10. Which of the following best describes your employment status currently? S1q9
    1. Full-time employment
    2. Part-time/casual employment
    3. Retired
    4. Household/carer duties
    5. Student
    6. Volunteer
    7. Unemployed/looking for work
    8. Unable to work
    9. Prefer not to say
    10. Other (please specify): _______________ s1q9a

**Please tell us a little bit about your cancer diagnosis Section_1_part_2**

1. What type of cancer were you diagnosed with? S1q10
   1. Breast
   2. Prostate
   3. Bowel
   4. Melanoma/skin cancer
   5. Lung
   6. Ovarian
   7. Other (please specify): _______________s1q10a
   8. I do not know
2. What type/s of treatment have you received (please select all that apply) s1q11
   1. Surgery
   2. Chemotherapy
   3. Radiotherapy
   4. Hormonal Therapy (e.g., Tamoxifen)
   5. Immunotherapy
   6. No active treatment – under observation/surveillance
   7. Other (please specify): _______________ s1q11a
   8. I do not know
3. What stage of the cancer journey are you currently in? s1q12
   ***NOTE: This question will form the basis of the SKIP LOGIC to assign groups.***
   1. I have just been diagnosed (have not started treatment)
   2. I am currently in active treatment
   3. I have just finished active treatment (within the last 3 months)
   4. I finished treatment over 3 months ago
   5. I finished treatment over 6 months ago
4. If known, what is the stage of your cancer? s1q13
   1. Stage 0: cancers are found in the place they started. They have not spread.
   2. Stage 1: the cancer has not grown deeply into nearby tissues. It has not spread to the lymph nodes or other parts of the body. It is often called early-stage cancer.
   3. Stage 2 & 3: cancers that have grown deeply into nearby tissue. It may have spread to lymph nodes but not to other parts of the body
   4. Stage 4: cancer has spread to other organs or parts of the body. It may be also called advanced or metastatic cancer.
   5. I’m not sure
   6. Not applicable
5. Have you ever visited or gotten help from a cancer survivorship centre? S1q14
   1. Yes
   2. No
   3. Maybe, but I am not sure if it was at a formal survivorship centre
   4. I do not know what a survivorship centre is
   5. I did not know NSW had any survivorship centres
6. Have you received your treatment within a private or public hospital setting? S1q15
   1. Public Hospital only
   2. Private Hospital only
   3. Both Public and Private Hospitals
   4. I am not sure
7. Which of the following topics are important to you? (Select all that apply) s1q16
   1. Diagnosis information (including staging)
   2. Treatment information
   3. Treatment side effects information
   4. Things you can do to help yourself get well
   5. Useful contacts (e.g., specialist, 24-hour emergency line)
   6. Psychological / emotional wellbeing
   7. Physical wellbeing
   8. Information needs
   9. Social wellbeing
   10. Spiritual/religious wellbeing
   11. Information for loved ones / carers
8. Now, in your own words, could you please tell us what you think cancer survivorship care should look like? S1q17

FREE TEXT

1. When do you think cancer survivorship care should begin? S1q18
   1. At diagnosis
   2. Before beginning treatment
   3. During treatment
   4. After completing treatment
2. **PARTICIPANTS WHO HAVE JUST BEEN DIAGNOSED OR IN ACTIVE TREATMENT**

**Please tell us about services you have accessed since your diagnosis. Section_2**

1. Since diagnosis, have you seen any of these health professionals for treatment: (select all that apply)? S2q1
   1. GP
   2. Specialists (oncologist)
   3. Palliative care specialist
   4. Pathologist
   5. Psychologist/Counsellor
   6. Dietician
   7. Exercise physiologist
   8. Speech pathologist
   9. Occupational therapist
   10. Care coordinator or nurse specialist
   11. Dentist
   12. Other: please specify s2q1a
   13. I have not seen any heath professional for follow up / care – SKIP LOGIC to below (s2q2)
2. Please tell us the reason(s) why this type of care was not provided to you? (Select all that apply) s2q2
   1. I have no need
   2. I do not know where to access services
   3. I did not know services were available around me
   4. They cost too much
   5. I do not have enough time
   6. I could not get an appointment
   7. I did not receive a referral from my treating doctor
   8. I live too far away to access these health professionals
   9. I did not think they were important
   10. Other: please specify s2q2a
3. Since diagnosis, have you had the **need** to see any other health professional but have been **unable to**? (SKIP LOGIC – yes go to s2q4 and s2q5, no go to s2q6) s2q3
   1. Yes
   2. No
4. Which health professionals have you had the need to see but were unable to? S2q4
   1. GP
   2. Specialists (oncologist)
   3. Palliative care specialist
   4. Pathologist
   5. Psychologist/Counsellor
   6. Dietitian
   7. Exercise physiologist
   8. Speech pathologist
   9. Occupational therapist
   10. Care coordinator or nurse specialist
   11. Dentist
   12. I’m not sure
   13. Other: please specify s2q4a
5. Why do you believe you were unable to access these health professionals? S2q5
   1. I do not know where to access services
   2. I did not know services were available around me
   3. They cost too much
   4. I do not have enough time
   5. I could not get an appointment
   6. I did not receive a referral from my treating doctor
   7. I live too far away to access these health professionals
   8. I did not think they were important
   9. Other: please specify s2q5a

***Supportive care compliments your medical care. It refers to all aspects of your health and wellbeing, including physical symptoms, family, social and practical needs, spiritual and religious needs, information needs and emotional and psychological needs. During your cancer journey, you may need support for some or all of these things.***

1. Did you receive any information about available supportive care services? S2q6
   1. Yes, from my hospital/treating team
   2. Yes, from my GP
   3. Yes, from family/friends
   4. I found information online
   5. I did not receive any information
   6. I did not know there was supportive care services for cancer patients
   7. Yes, other, please specify s2q6a
2. Overall, do you feel the treatment and support you received during your cancer journey met your needs? S2q7
   1. Yes
   2. No
   3. I am not sure
3. Were you aware of **HOW** to seek help for a symptom or side effect during the course of your cancer journey? S2q8
   1. Yes, my health team told me who to contact and how to contact them
   2. No, I had to use my known knowledge or search for answers when symptoms/side effects arose
   3. I never had a need to seek help for symptoms/side effects
4. Were you aware of **WHEN** to seek help for a symptom or side effect during the course of your cancer journey? S2q9
   1. Yes, my health team told me what to look out for and when
   2. No, I had to use my known knowledge or search for answers when symptoms/side effects arose
   3. I never had a need to seek help for symptoms/side effects
5. Have you ever accessed any supportive care services? (SKIP LOGIC, Yes go to s2q11, s2q12 and s2q13, no go to s2q14) s2q10
   1. Yes
   2. No
   3. Not yet, but maybe in the future
6. If yes, which supportive care services have you accessed? (Select all that apply) s2q11
   1. Dietitian
   2. Lymphoedema practitioners
   3. Music or art therapists
   4. Occupational therapists
   5. Exercise physiologists
   6. Pastoral care workers
   7. Physiotherapists
   8. Psychiatrists
   9. Psychologists
   10. Social workers
   11. Speech and language therapists
   12. Other, please specify: ______________ s2q11a
7. How easy was it to access supportive care services during your cancer journey (e.g., access to exercise classes, psychologists, dietitians etc.)? – SKIP LOGIC (if neither/I did not need access then go to s2q14 if all other answers then go to s2q13) s2q12
   1. Very easy
   2. Easy
   3. Neither / I did not need to access the service
   4. A little difficult
   5. Very difficult
8. Who referred you to the supportive care services? (select all) – SKIP LOGIC (go to next question) s2q13
   1. Hospital or treatment centre
   2. Specialist (oncologist/surgeon)
   3. GP
   4. Care coordinator or nurse coordinator
   5. Allied health professional (counsellor, psychologist)
   6. Self-referral
   7. Other, please specify: ______________ s2q13a

***A survivorship care plan is a digital or written document from your health professional that includes information about your cancer treatment and what you can do to look after yourself. It is likely to include a summary of your treatment/s, follow-up care information and a wellness plan to help you make healthy living changes. Other health professionals can use the plan to help organise your care.***

1. Did you receive a written or digital survivorship care plan from your health professional? – SKIP LOGIC (if yes then go to s2q15, s2q16, s2q17, s2q18, and s2q19, no then go to s2q20)s2q14
   1. Yes
   2. No
   3. I am not sure
2. If yes, when did you receive your care plan? – SKIP LOGIC (go to next) s2q15
   1. At diagnosis
   2. Before beginning treatment
   3. During treatment
   4. After finishing treatment
3. Who created this care plan for you? – SKIP LOGIC (go to next) s2q16
   1. Specialist (oncologist, surgeon)
   2. GP
   3. Care coordinator or nurse specialist
   4. Allied health professional (counsellor, dietitian)
   5. Other, please specify: ______________ s2q16a
4. Did your survivorship care plan include any of the following? (select all) s2q17
   1. A summary of your treatment/s
   2. Follow-up care information (appointments, tests, scans, long-term side effects)
   3. Wellness plan/information (healthy diet, coping with emotions, exercise)
   4. Other: please specify: ______________ s2q17a
5. Did your care plan include information on any of these additional topics? (Select all) s2q18
   1. Physical concerns and issues (e.g., pain, feeling sick, tiredness)
   2. Psychological concerns and issues (e.g., fear, worry, anxiety)
   3. Social concerns and issues (e.g., childcare, financial issues, home help)
   4. Cultural concerns and issues (e.g., maintaining relationships and community)
   5. Informational concerns and issues (e.g., where to access services, who to call with questions or about symptoms)
   6. Spiritual concerns and issues (e.g., hope, courage, faith)

Other, please specify: ______________ s2q18a

1. Before you were given your care plan, did your health care professional ask about your needs, concerns, and opinions about managing your cancer and living well after treatment? – SKIP LOGIC (go to next) s2q19
   1. Yes, my concerns were discussed, and they helped shape my plan’s development
   2. Yes, my concerns were discussed but they were not reflected in my plan’s development
   3. No, my concerns were not discussed, and I did not have the chance to help shape my plan’s development
   4. No, I had no concerns to discuss

**Barriers and facilitators of optimal survivorship care.**

1. At what point did your health team talk to you about ongoing **physical** care during your cancer journey? (Select all) s2q20
   *Note: physical needs may include short/long-term side effects such as weight loss/gain, pain, tiredness, shortness of breath.*
   1. At diagnosis
   2. Before beginning treatment
   3. During treatment
   4. After completing treatment
   5. Never
2. At what point did your health team talk to you about ongoing **psychological** care during your cancer journey? (Select all) s2q21
   *Note: psychological needs may include mental wellbeing and relationships, fear of cancer recurrence, disease progression or feelings such as anger, anxiety, depression, or sadness.*
3. At diagnosis
4. Before beginning treatment
5. During treatment
6. After completing treatment
7. Never
8. At what point did your health team talk to you about ongoing **social (including educational, financial, and occupational issues)** care during your cancer journey? (Select all) s2q22
   *Note: social needs may include your issues relating to education, finances, or work.*
9. At diagnosis
10. Before beginning treatment
11. During treatment
12. After completing treatment
13. Never
14. At what point did your health team talk to you about ongoing **cultural** care during your cancer journey? (Select all) s2q23
    *Note: cultural needs may include maintaining relationships with others, and cultural identity / where you fit in society.*
15. At diagnosis
16. Before beginning treatment
17. During treatment
18. After completing treatment
19. Never
20. At what point did your health team talk to you about your **informational needs** during your cancer journey? (Select all) s2q24
    *Note: information needs may include ability to access good health information, find reliable information about your diagnosis and treatment and find what support services are available.*
21. At diagnosis
22. Before beginning treatment
23. During treatment
24. After completing treatment
25. Never
26. At what point did your health team talk to you about ongoing **spiritual** care during your cancer journey? (Select all) s2q25

*Note: spiritual needs may include issues related to your beliefs, changing sense of self due to diagnosis or concerns related to aspects such as feelings of guilt/shame.*

1. At diagnosis
2. Before beginning treatment
3. During treatment
4. After completing treatment
5. Never
6. What, if anything, do you believe is missing from ongoing supportive care for cancer patients? S2q26

FREE TEXT

1. What is most important to you in terms of ongoing support and care while you live with and beyond cancer? S2q27

FREE TEXT

Additional questions

**Would you be interested in sharing your survivorship story with the research team at Cancer Council NSW? SKIP LOGIC – yes go to next**

**Could you please provide us with a phone number and email for us to get in touch?**

**Phone number:**

**Email address:**

1. **PARTICIPANTS WHO HAVE COMPLETED ACTIVE TREATMENT**

**Please tell us about services you have accessed since finishing active treatment. Section_3**

***A survivorship care plan is a digital or written document from your health professional that includes information about your cancer treatment and what you can do to look after yourself. It is likely to include a summary of your treatment/s, follow-up care information and a wellness plan to help you make healthy living changes. Other health professionals can use the plan to help organise your care.***

1. Did you receive a written or digital survivorship care plan from your health professional? – SKIP LOGIC (yes go to s3q2, s3q3, s3q4, s3q5, s3q6, and s3q7, no or I am not sure then go to s3q8) s3q1
   1. Yes
   2. No
   3. I am not sure
2. If yes, when did you receive your care plan? – SKIP LOGIC (go to next) s3q2
   1. At diagnosis
   2. Before beginning treatment
   3. During treatment
   4. After finishing treatment

1. Who created this care plan for you? – SKIP LOGIC (go to next) s3q3
   1. Specialist (oncologist, surgeon)
   2. GP
   3. Care coordinator or nurse specialist
   4. Allied health professional (counsellor, dietitian)
   5. Other, please specify: ______________ s3q3a
2. Did your survivorship care plan include any of the following? (select all) s3q4
   1. A summary of your diagnosis and treatment/s
   2. Follow-up care information (appointments, tests, scans, long-term side effects)
   3. Wellness plan/information (healthy diet, coping with emotions, exercise)
   4. Other: please specify: ______________ s3q4a
3. Did your care plan include information on any of these additional topics? (Select all) s3q5
   1. Physical concerns and issues (e.g., pain, feeling sick, tiredness)
   2. Psychological concerns and issues (e.g., fear, worry, anxiety)
   3. Social concerns and issues (e.g., childcare, financial issues, home help)
   4. Cultural concerns and issues (e.g., maintaining relationships and community)
   5. Informational concerns and issues (e.g., where to access services, who to call with questions or about symptoms)
   6. Spiritual concerns and issues (e.g., hope, courage, faith)
   7. Other, please specify: ______________ s3q5a
4. Before you were given your care plan, did your health care professional ask about your needs, concerns, and opinions about managing your cancer and living well after treatment? – SKIP LOGIC (go to next) s3q6
   1. Yes, my concerns were discussed, and they helped shape my plan’s development
   2. Yes, my concerns were discussed but they were not reflected in my plan’s development
   3. No, my concerns were not discussed, and I did not have the chance to help shape my plan’s development
   4. No, I had no concerns to discuss
5. Since stopping active treatment, has your care plan been updated or reviewed? S3q7
6. Yes, once
7. Yes, more than once
8. No, never
9. I am not sure
10. After finishing treatment, do you know what side effects or changes in your health to look out for that may be associated with your cancer? S3q8
11. Yes, I was given information by my treatment team (oncologist, surgeon etc.)
12. Yes, I was given information by my GP
13. Yes, I found my own information from a source I trust
14. No, I do not know what to look out for
15. Since your cancer diagnosis, have you been advised to undertake any screening/tests for other cancers? S3q9
16. Yes, I was given information by my treatment team (oncologist, surgeon etc.)
17. Yes, I was given information by my GP
18. Yes, I found my own information from a source I trust
19. No, I did not know this was necessary
20. Have you been provided with any information about how to reduce your risk of cancer recurrence or other cancers (e.g., maintaining a healthy diet/exercise program, quitting smoking, reducing/stopping alcohol, maintaining a healthy weight)? S3q10
21. Yes, from my specialist (oncologist)
22. Yes, from my broader specialist treatment team (nurse coordinator, clinical nurse specialist)
23. Yes, from my GP
24. Yes, from an allied health professional (e.g., psychologist, exercise physiologist)
25. No, I never received this type of information
26. I’m unsure if I have received this type of information
27. Other, please specify: ___________________ s3q10a
28. Since completing treatment, have you seen any of these health professionals for treatment: (select all that apply)?s3q11
    1. GP
    2. Specialists (oncologist)
    3. Palliative care specialist
    4. Pathologist
    5. Psychologist/Counsellor
    6. Dietitian
    7. Exercise physiologist
    8. Speech pathologist
    9. Occupational therapist
    10. Care coordinator or nurse specialist
    11. Dentist
    12. Other: please specify s3q11a
    13. I have not seen any heath professional for follow up / care – SKIP LOGIC to below (go to s3q12, all other answers go to s3q13)
29. Please tell us the reason(s) why this type of care was not provided to you? (Select all that apply) s3q12
    1. I have no need
    2. I do not know where to access services
    3. I did not know services were available around me
    4. They cost too much
    5. I do not have enough time
    6. I could not get an appointment
    7. I did not receive a referral from my treating doctor
    8. I live too far away to access these health professionals
    9. I did not think they were important
    10. Other: please specify s3q12a (does not come up)
30. Since completing treatment, have you had the **need** to see any other health professional but have been **unable to**? (SKIP LOGIC – yes go to s3q14, no go to s3q16 – currently taking to s3q15) s3q13
    1. Yes
    2. No
31. Which health professionals have you had the need to see but were unable to? S3q14
    1. GP
    2. Specialists (oncologist)
    3. Palliative care specialist
    4. Pathologist
    5. Psychologist/Counsellor
    6. Dietician
    7. Exercise physiologist
    8. Speech pathologist
    9. Occupational therapist
    10. Care coordinator or nurse specialist
    11. Dentist
    12. I’m not sure
    13. Other: please specify s3q14a
32. Why do you believe you were unable to access these health professionals? S3q15
    1. I do not know where to access services
    2. I did not know services were available around me
    3. They cost too much
    4. I do not have enough time
    5. I could not get an appointment
    6. I did not receive a referral from my treating doctor
    7. I live too far away to access these health professionals
    8. I did not think they were important
    9. Other: please specify s3q15a
33. How effective do you think care for cancer patients is after they finish active treatment? S3q16
34. Extremely effective
35. Very effective
36. Moderately effective
37. Slightly effective
38. Not at all effective

**Exploring survivorship care. Section_3_part_2**

1. Where do you go for information related to your cancer journey? (select all that apply) s3q17
2. Online
3. Face-to-face with specialist team (oncologist, surgeon etc.)
4. Allied health professionals (care coordinators, psychologists etc.)
5. Loved ones
6. Other: please specify (doesn’t show up) s3q17a
7. Do you believe this is the most trustworthy source of information? S3q18
   - 1. Yes, that is why I use this source/s
     2. No, but it’s the only source/s I have available
     3. I’m not sure if the sources are trustworthy

***Supportive care compliments your medical care. It refers to all aspects of your health and wellbeing, including physical symptoms, family, social and practical needs, spiritual and religious needs, information needs and emotional and psychological needs. During your cancer journey, you may need support for some or all of these things.***

1. Did you receive any information about available supportive care services? S3q19
2. Yes, from my hospital/treating team
3. Yes, from my GP
4. Yes, from family/friends
5. I found information online
6. I did not receive any information
7. I did not know there was supportive care services for cancer patients
8. Overall, do you feel the treatment and support you received during your cancer journey met your needs? S3q20
9. Yes
10. No
11. I am not sure
12. How easy was it to access supportive care services during your cancer journey (e.g., access to exercise classes, psychologists, dieticians etc.)? – SKIP LOGIC (if neither/I did not need to access then go to s3q23. All other answers go to s3q22) s3q21
13. Very easy
14. Easy
15. Neither / I did not need to access the service
16. A little difficult
17. Very difficult
18. Where did you go for help? (Select all) – SKIP LOGIC s3q22
19. Hospital or treatment centre
20. Specialist (oncologist/surgeon)
21. GP
22. Care coordinator or nurse coordinator
23. Allied health professional (counsellor, psychologist)
24. Other: please specify s3q22a

**Barriers and facilitators of optimal survivorship care. Section_3_part_3**

1. At what point did your health team talk to you about ongoing **physical** care during your cancer journey? (Select all) s3q23
   *Note: physical needs may include short/long-term side effects such as weight loss/gain, pain, tiredness, shortness of breath.*
2. At diagnosis
3. Before beginning treatment
4. During treatment
5. After completing treatment
6. Never
7. At what point did your health team talk to you about ongoing **psychological** care during your cancer journey? (Select all) s3q24
   *Note: psychological needs may include mental wellbeing and relationships, fear of cancer recurrence, disease progression or feelings such as anger, anxiety, depression, or sadness.*
   1. At diagnosis
   2. Before beginning treatment
   3. During treatment
   4. After completing treatment
   5. Never
8. At what point did your health team talk to you about ongoing **social (including educational, financial, and occupational issues)** care during your cancer journey? (Select all) s3q25
   *Note: social needs may include your issues relating to education, finances, or work.*
9. At diagnosis
10. Before beginning treatment
11. During treatment
12. After completing treatment
13. Never
14. At what point did your health team talk to you about ongoing **cultural** care during your cancer journey? (Select all) s3q26
    *Note: cultural needs may include maintaining relationships with others, and cultural identity / where you fit in society.*
15. At diagnosis
16. Before beginning treatment
17. During treatment
18. After completing treatment
19. Never
20. At what point did your health team talk to you about your **informational needs** during your cancer journey? (Select all) s3q27
    *Note: information needs may include ability to access good health information, find reliable information about your diagnosis and treatment and find what support services are available.*
21. At diagnosis
22. Before beginning treatment
23. During treatment
24. After completing treatment
25. Never
26. At what point did your health team talk to you about ongoing **spiritual** care during your cancer journey? (Select all) s3q28

*Note: spiritual needs may include issues related to your beliefs, changing sense of self due to diagnosis or concerns related to aspects such as feelings of guilt/shame.*

1. At diagnosis
2. Before beginning treatment
3. During treatment
4. After completing treatment
5. Never
6. What, if anything, do you believe is missing from ongoing supportive care for cancer patients? S3q29

FREE TEXT

1. What is most important to you in terms of ongoing support and care while you live with and beyond cancer? S3q30

FREE TEXT

1. Would you be interested in sharing your survivorship story with the research team at Cancer Council NSW? Sharingstory – SKIP LOGIC
   1. Yes
   2. No
2. If yes, could you please provide us with a phone number and email for us to get in touch phoneandnumber
   1. Phone Number: phonenumber
   2. Email: emailaddress

**ALL PARTICIPANTS TO SEE**

**Thank you for taking the time to complete this questionnaire.**

**If you have any questions about participating in this research project, please get in touch with Jessica Sheppard, on**[**+61293080240**](tel:+61293080240)**or at**[**jessica.sheppard@nswcc.org.au**](mailto:jessica.sheppard@nswcc.org.au)

**Please press submit to complete the form.**

**The Cancer Council NSW helpline can provide you with information or support if this questionnaire has raised issues you would like to discuss. Please call 13 11 20.**
